# Perceptions of Research Infrastructure and Support in Northern New England

**DOI:** 10.46804/2641-2225.1257

**Published:** 2026-05-18

**Authors:** Carolyn E. Gray, Kathryn Bizier, Brenda M. Joly

**Affiliations:** a Catherine Cutler Institute, Muskie School of Public Service, University of Southern Maine, Portland, Maine; b Public Health Program, Muskie School of Public Service, University of Southern Maine, Portland, Maine

**Keywords:** Program evaluation, Support of research, Organization and administration, Research institutes

## 1. Introduction

The Northern New England Clinical and Translational Research Network (NNE-CTR) was established in 2017,^1,2^ to bring together researchers and the community in Maine, Vermont, and New Hampshire to address health care needs and build research infrastructure in the region. CTR programs are intended to build the research infrastructure and workforce in states with reduced clinical capacity.^3^ As part of this work, they routinely assess researcher needs and perceptions.^4–6^ In Maine, the NNE-CTR Tracking and Evaluation Core developed and administered a survey to assess the current research infrastructure, the role of the NNE-CTR, research barriers, and suggestions for improvement.

## 2. Methods

A voluntary 13-item online survey was sent to 579 NNE-CTR active registrants in March 2025 using Qualtrics. Registrants were excluded from the survey if they were considered inactive; reported working in states other than Maine, Vermont, or New Hampshire; served as a current or past NNE-CTR core lead; or were a member of the Administrative Core and Tracking and Evaluation Core. CTR registrant data were used for demographics; no identifying demographic data were reported in the results.

Univariate frequency statistics were run for quantitative survey items. Open-ended responses were coded by 2 evaluation staff. Each response was coded using an inductive approach with thematic analysis. Staff reviewed codes to confirm categories and coding of responses. Bivariate analyses were conducted to compare the demographics of respondents with NNE-CTR registrants (n = 480) and assess if respondents were representative of the NNE-CTR.

## 3. Results

**Fig. 1.**
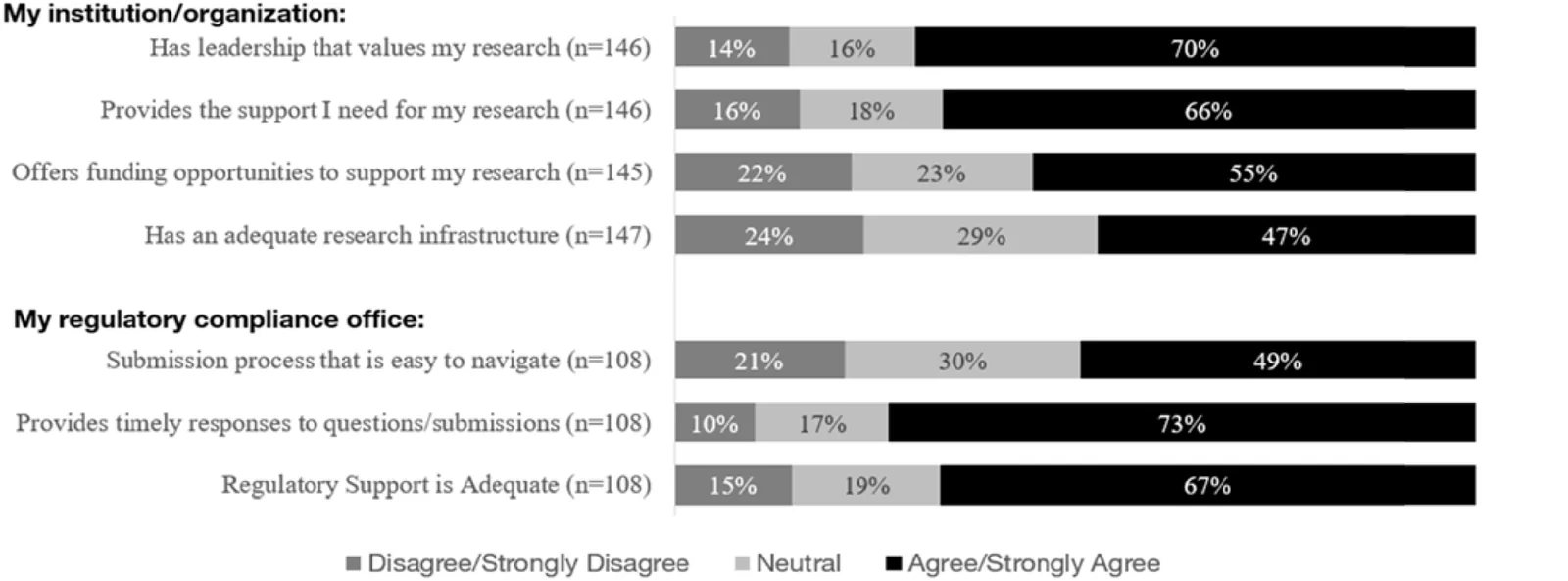
Component Bar graph showing respondents’ level of agreement with institutional environment and regulatory process. Data source: NNE-CTR registrant survey.

The survey had a 26% (148 of 579) response rate, and most respondents were working in Maine (Maine: 59% [88 of 148], Vermont: 33% [49 of 148], New Hampshire: 7% [11 of 148]). Approximately two-thirds of survey participants reported that their institution valued their research (70% [102 of 146]) and supported their work (66% [97 of 146]). Approximately half of respondents reported provision of institutional funding opportunities (55% [80 of 145]) and adequate research infrastructure (47% [69 of 147]) (Fig. 1).

Respondents had varied experience with submitting regulatory compliance applications as a principal investigator (or co-investigator) in the past 2 years. Approximately one quarter (26% [38 of 146]) reported no applications, whereas almost half (49% [71 of 146]) reported between 1 and 3 applications. For those with at least 1 application, about half (49% [53 of 108]) reported that the regulatory submissions were easy. About two-thirds (73% [79 of 108]) reported that responses from the regulatory boards were timely and support for regulatory submissions was adequate (67% [72 of 108]) (Fig. 1). Less than a quarter of respondents had 1% to 25% of designated time for research (19% [21 of 110]).

In terms of respondent characteristics, most respondents were female (63% [93 of 148]), followed by male (36% [54 of 148], and non-binary (data suppressed due to 5 or fewer respondents). Most respondents were non-Hispanic/Latinx (95% [121 of 127]), followed by Hispanic/Latinx, reporting “don’t know”, and “prefer not to answer” (data suppressed due to 5 or fewer respondents). Although the majority reported being White (86% [113 of 132]), 6% [8 of 132]) reported being Asian, 6% (8 of 132) reported more than 1 race, followed by Black or African American, and American Indian or Alaskan Native (data suppressed due to 5 or fewer respondents) (Table 1). Survey respondents were similar in characteristics when comparing their registration information to the NNE-CTR registration, with the exception of state. Respondents were significantly different than overall registrants in terms of state (*P* = .007); more survey respondents were working in Maine.

The survey asked about how the NNE-CTR has helped researchers, as well as the barriers to research and suggestions for improvement. Table 2 provides a summary of the top 7 themes based on what helped, barriers encountered, and suggestions.

### 3.1. What has helped

The most common theme for how the NNE-CTR supported researchers was through funding, followed by providing an opportunity for collaboration/networking, and training/education. One respondent stated that the NNE-CTR “provided a resource to ask questions, get research support, and potentially provide funding and collaborations.”

### 3.2. Barriers encountered

The top barriers included funding, process/infrastructure issues (eg, interconnected systems with different and changing policies, need for streamlined processes), and staffing time/support.

### 3.3. Suggestions

Financial support was the primary suggestion for addressing research barriers, followed by having support for hiring/staffing and data analytics/navigation. Protected time and collaboration/networking were also mentioned but not as prominent.

## 4. Discussion

Our survey response rate was similar to other CTR-related online surveys.^4,6^ Results indicated a fairly representative sample of NNE-CTR members who participated in this study. Overall, survey participants reported positive perceptions about the research infrastructure and supports in place. Yet, there are several identified areas for growth. As the NNE-CTR continues to expand its efforts, this information can help direct future efforts to support clinical and translational researchers. Further exploration to learn more about what has helped, barriers encountered, and suggestions for improvement could be investigated through interviews or focus groups to inform future efforts and potentially assess changes over time. Expanded qualitative approaches have been used to successfully study barriers and facilitators among new investigators,^7–9^ and similar efforts would likely provide further insight to guide the NNE-CTR.

## 5. Conclusions

Surveying NNE-CTR registrants about their perceptions of research infrastructure, how the initiative has supported researchers, barriers encountered, and suggestions for improvement are important to inform ongoing efforts. Continued investment is needed to enhance support for clinical and translational researchers, and to stimulate novel research endeavors in Northern New England.

## Figures and Tables

**Table 1.** Participant characteristics compared with NNE-CTR registrants^a^.

Characteristic	NNE-CTR Registrants No. (%)	Survey Respondents No. (%)
State^b^	n = 480	n = 148
Maine	253 (53)	88 (59)
New Hampshire	16 (3)	11 (7)
Vermont	194 (40)	49 (33)
Other	17 (4)	NA
Race	n = 412	n = 132
American Indian or Alaskan Native	DS	DS
Asian	42 (10)	8 (6)
Black or African American	12 (3)	DS
White	331 (80)	113 (86)
More than one race	11 (3)	8 (6)
Don’t know	DS	0 (0)
Prefer not to answer	14 (3)	0 (0)
Ethnicity	n = 395	n = 127
Hispanic/Latinx	7 (2)	DS
Not Hispanic/Latinx	367 (93)	121 (95)
Prefer not to report	14 (4)	DS
Don’t know	7 (2)	DS
Gender	n = 480	n = 148
Female	291 (61)	93 (63)
Male	178 (37)	54 (36)
Non-binary	DS	DS
Other	DS	0 (0)
Prefer not to answer	6 (1)	0 (0)

Abbreviations: DS, data suppressed due to 5 or fewer respondents; NA, not applicable, NNE-CTR, Northern New England Clinical and Translational Research Network.

a Data Source: NNE-CTR registration.

b *P* < .05.

**Table 2.** Top 7 themes identified in each category (n = 148)^a^.

Theme	No (%)
What helped	
Pilot/funding support	35 (24)
Network, connections, collaborations	26 (18)
Training, webinars, information, Project ECHO	14 (10)
Other	13 (9)
Research navigator support, grant support	10 (7)
Technology/research equipment	9 (6)
Career support	9 (6)
Barriers encountered	
Money/funding	31 (21)
Other	28 (19)
Process/infrastructure	26 (18)
Full time staffing/staff support	21 (14)
IRB-related	16 (11)
Protected time	12 (8)
Time	11 (7)
Suggestions	
Financial support	29 (20)
Other	28 (19)
Hiring/staffing to help address research and/or academic demands	21 (14)
Navigation and data analytics	17 (11)
Time, protected time	17 (11)
Collaboration and networking opportunities	15 (10)
Technology	12 (8)

Abbreviations: ECHO, Extension for Community Healthcare Outcomes; IRB, institutional review board; NNE-CTR, Northern New England Clinical and Translational Research Network.

a Data source: NNE-CTR registrant survey.
